# Screening for adrenal suppression in children with inflammatory bowel disease discontinuing glucocorticoid therapy

**DOI:** 10.1186/1471-230X-14-51

**Published:** 2014-03-24

**Authors:** Marianne Sidoroff, Kaija-Leena Kolho

**Affiliations:** 1 Children’s Hospital, University of Helsinki and Helsinki University Central Hospital, Helsinki FI-00029, Finland

**Keywords:** Adverse effects, Crohn’s disease, Paediatrics, Steroids, Ulcerative colitis

## Abstract

**Background:**

Pharmacological doses of corticoids may result in adrenal suppression but with individual sensitivity. In paediatric inflammatory bowel disease (IBD), glucocorticoids are needed in the majority of the patients but there are less studies related to tapering off the drugs. The objective of this study was to estimate the frequency of adrenal insufficiency in children with IBD that were at the end of their systemic glucocorticoid therapy course.

**Methods:**

The study was a retrospective case series of 59 consecutive paediatric IBD patients (median age 14.1 years; Crohn’s disease n = 22, ulcerative colitis n = 26, unclassified colitis n = 11) that were on oral prednisolone therapy about to be discontinued. The study patients were treated in a tertiary university hospital setting. Serum morning cortisol was measured with Immulite 2000 cortisol kit. Values < 20 nmol/l are undetectable and indicate adrenal suppression, values > 69 nmol/l are considered to represent normal basal secretion.

**Results:**

The morning cortisol was below the reference range in 20% of the patients and undetectable in 10%. Low cortisol levels associated with higher daily glucocorticoid doses (median 7.2 mg/m^2^ vs. 3.0 mg/m^2^ in patients with normal cortisol levels, p < 0.05) and with the long duration of the treatment (median 11 months vs. 4 months, p < 0.05). Patients with undetectable cortisol levels recovered within few weeks (median 5.6 weeks).

**Conclusions:**

In paediatric IBD prolonged courses of glucocorticoids are frequent due to the steroid-dependent nature of the disease in a considerable proportion of patients. Adrenal suppression may occur in at least one fifth of the patients despite slowly tapering off the glucocorticoids. Notably, this is based on a set of serum cortisol measurements by request of experienced clinicians. All paediatric IBD patients receiving conventional doses of oral glucocorticoids should be subjected to screening for adrenal suppression when anticipated discontinuation of the drug.

## Background

Inflammatory bowel disease (IBD) encompassing ulcerative colitis (UC), Crohn’s disease (CD) and unclassified colitis (IBDU) is a chronic, disabling disease of the gastrointestinal tract. Similarly to adult patients, the number of children suffering from IBD has risen during the past decades and in Western countries its incidence is growing with an alarming pace [1-4]. The reason for this increase is at present unknown.

Glucocorticoids represent the backbone of treatment of active IBD [5-7]. In paediatric patients the therapy is commenced with 1-2 mg/kg/day of prednisone equivalents (max 60 mg) and tapered off preferably during 10 weeks [6]. However, glucocorticoid dependency affects approximately 20-40% of the patients and prolonged steroid courses are often seen in clinical practice [8,9].

Long-term supraphysiological dosing of systemic glucocorticoids produces side effects that vary in presentation from patient to patient. Adrenal suppression is a condition in which adrenal glands do not produce adequate amounts of cortisol in response to physiological stress. It is caused by the suppression of the hypothalamic-pituitary-adrenal (HPA) axis by the circulating exogenous glucocorticoids and may lead into adrenal crisis or even death [10].

The measurement of the first morning cortisol is recommended as a useful first step to screen cases of suspected adrenal insufficiency [11]. If the levels are low or undetectable, the patient most likely suffers from adrenal suppression. However, if the first morning cortisol levels are normal, the patient may still have a blunted HPA axis response to stress. Then the ACTH stimulation test should follow in order to confirm the responsiveness of the HPA axis [12].

Risk factors for the development of adrenal suppression are not yet fully understood. However, high glucocorticoid dose and long duration of the therapy seem to associate with the increased risk [10] but intestinal absorption and genetic factors related to glucocorticoid sensitivity are likely to play an additional role [13]. Indeed, it is recommended that all children who have received pharmacologic doses of systemic glucocorticoid therapy for more than two weeks should be assessed for adrenal insufficiency [14]. Paediatric patients with active IBD on oral glucocorticoid therapy fulfil the at-risk criteria, however in many of them the condition goes unnoticed [12]. Adrenal suppression is an understudied and possibly an under-recognized complication of extended steroid use in paediatric IBD patients with potentially lethal consequences. We therefore aimed to assess the frequency of adrenal insufficiency in children with IBD receiving systemic glucocorticoids.

## Methods

The study group consisted of paediatric IBD patients that were treated in the Outpatient Clinic of the Children’s Hospital in Helsinki University Central Hospital, a tertiary care center, between January 2008 and July 2011. All diagnoses were based on modern endoscopic criteria including upper gastrointestinal endoscopy and imaging of the small bowel when there is a suspicion of Crohn’s disease [15]. The majority of our patients with CD and UC need glucocorticoids, the proportion of such patients being stable regardless of the year of diagnosis [16]. Experienced gastroenterologists instructed the tapering of glucocorticoids according to disease activity along the lines suggested in the recent paediatric guidelines of UC [17]. The data was collected retrospectively. The inclusion criteria were 1) IBD diagnosis [15] and 2) serum cortisol measured when oral predisolone was about to be discontinued. The serum cortisol was measured by request of clinicians. Altogether, 59 patients were identified. The clinical characteristics of the patients are presented in Table 1. Steroid therapy related side effects (cushingoid rounding of the cheeks, weight gain, acne, mood changes) were carefully registered. Serum cortisol was measured routinely with Immulite 2000 cortisol kit (Diagnostic Products Corporation, LA, CA) in samples scheduled at 9 AM [18]. The daily prednisolone dose was withheld until after the measurement. The detection limit of the assay is 20 nmol/l, intra-assay coefficient of variation of <7.4% and inter-assay coefficient of variation of < 9.55%. The reference range for normal serum morning cortisol defined by the clinical laboratory is 69-632 nmol/l for children aged 2-13 years, 69-789 nmol/l for children aged 14-15 years and 150-650 nmol/l for patients older than 16 years.

**Table 1:** The clinical characteristics of the 59 paediatric patients with inflammatory bowel disease (IBD)

Diagnosis CD/IBDU/UC*	**22/11/26**
Male/female	29/30
Age at the time of the study (median, range)	14 yrs (2.6–18)
Prednisolone daily dose	
10 mg	21
5 mg	18
5 mg on alternate days	20
Length of the prednisolone therapy (months; median, range)	5 (0.5–39)
Maintenance medication**	
5-ASA	27
5-ASA and/or Aza	19
TNF-α-antagonist	11
No medication	2

The first morning cortisol level was analyzed during weaning or at the end of oral prednisolone therapy. *CD: Crohn’s disease; IBDU: unclassified colitis; UC: ulcerative colitis. **5-ASA: 5-acetosalicylic acid; Aza: azathioprine.

*P* < 0.05 was set for statistical significance. Mann-Whitney U-test, Kruskal-Wallis test and Spearman’s rank order correlation tests were used when appropriate. Values are expressed as median, range.

All patients were participants of our ongoing study on paediatric IBD approved by the ethics committee of the Children’s Hospital, University of Helsinki. The study was conducted in accordance with the ethical standards of the responsible committee on human experimentation and with the Helsinki Declaration of 1975, as revised in 1983. Unfortunately, the total number of paediatric IBD patients treated with glucocorticoids at our clinic during the study period was not registered.

## Results

**Figure 1:**
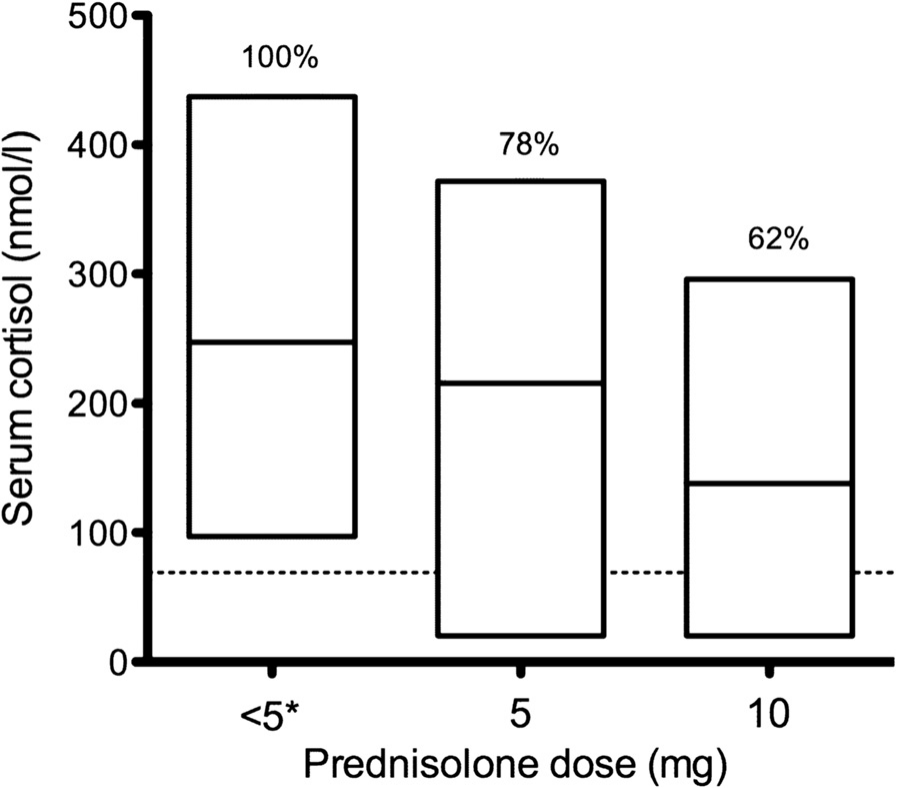
**Serum morning cortisol levels in paediatric IBD patients at the end of oral prednisolone therapy.** The 59 patients are divided into groups according to their present glucocorticoid dose. Bars represent the range of the cortisol level; horizontal lines the median cortisol level. The percentages above the bars describe how many percent of the patients had normal serum cortisol level. The horizontal dashed line at 69 nmol/l is the lower limit for normal serum morning cortisol level. In the first group receiving * <5 mg defines group of patients receiving 5 mg prednisolone on alternate days N = 20, in the second group receiving 5 mg a day N = 18, and in the third group receiving 10 mg a day N = 21.

At the end of the systemic prednisolone therapy when tapering off was considered, the median dose of prednisolone was 5 mg (<5 mg – 10 mg; median duration of the therapy 4.7 months; 0.5 – 39 months), Table 1. The median serum morning cortisol level was 204 nmol/l (<20-437 nmol/l) being below the reference range in 12/59 (20%) of the patients and undetectable (<20 nmol/l) in 6/12 patients (2/6 with CD; cortisol levels measured at 9.30 at latest). The children in whom the serum cortisol level was low received higher daily doses of prednisolone than patients that had normal cortisol level (median 7.2 mg/m^2^, range 5.4-10.2 mg/m^2^ vs. 3.0 mg/m^2^, 1.3-10 mg/m^2^, p < 0.05), Figure 1. In addition, the length of the steroid therapy was longer in the patients that had low cortisol levels (11 months, 3-39 months vs. 4 months, 0.5-33 months, p < 0.05). The dose of steroid was unaltered at minimum during the preceding week of cortisol measurement. Also previous steroid therapy affected the cortisol levels, patients that had received glucocorticoids before had lower cortisol levels than patients that were on their first steroid course (174 nmol/l, <20-319 nmol/l, n = 25 vs. 225 nmol/l, <20-437 nmol/l, n = 34, p < 0.05).

Disease subtype or medication was not associated with the morning cortisol levels (data not shown). Gender did not relate to the cortisol levels, however age correlated strongly (r = 0.421, p < 0.05), younger children having lower levels. The appearance of visible steroid therapy related side effects was not associated with the serum cortisol values. The time of measurement (median 8:39 AM, 6:20 – 10:42 AM) did not differ between the groups with normal vs. abnormal cortisol values (P = NS, data not shown). Reassuringly, patients with undetectable cortisol levels recovered within few weeks (5.6 weeks) when on hydrocortisone substitution or on low dose prednisolone/budesonide (and on alternate days on hydrocortisone). The cortisol measurements during recovery phase, however, occurred at 2 to 4- week intervals as scheduled by the clinicians. ACTH-stimulation tests are not routinely conducted at our clinic when discontinuing glucocorticoids in patients with IBD. The test was performed only in five of the study patients showing normal stimulatory response in all of them (data not shown).

## Discussion

In this pilot study we assessed serum morning cortisol levels in children with IBD at the end of systemic glucocorticoid (prednisolone) therapy. We found that in 20% of the patients the morning cortisol measurement was below the reference range and low cortisol values associated with higher daily glucocorticoid doses and longer duration of the therapy.

Systemic glucocorticoid therapy is a well known cause of secondary adrenal insufficiency. In IBD, 40 to 80 percent of the patients may receive steroid therapy at some point during the disease course (with a starting dose of 1-2 mg/kg), and several patients need prolonged courses because of steroid-dependent disease [8,16,19]. However, studies concerning HPA axis function in paediatric IBD patients are few. Escher *et al* found that children who received 10-20 mg of prednisone for active IBD had a mean morning plasma cortisol of 98 nmol/l [20]. When the dose was reduced to 2.5 – 10 mg that corresponds with the steroid doses used in our study, the mean serum morning cortisol level was similar to our results. However, they did not report how many patients failed their cutoff of 150 nmol/l for normal serum morning cortisol. In a study on adult IBD patients, the serum morning cortisol levels corresponded with our findings and also the number (29%) that had abnormal serum morning cortisol was roughly similar to ours [21].

Long duration of glucocorticoid therapy and high steroid doses are risk factors for the development adrenal insufficiency [10,14]. Consistently, in our study higher glucocorticoid doses and prolonged duration of the therapy associated with low serum morning cortisol levels. Patients were also more likely to have low serum morning cortisol levels if they had received previous steroid treatment. This is in concordance with a study on adult IBD patients, where a past history of glucocorticoid treatment was predictive of abnormal adrenal stimulation test [12]. Of notice, very low levels were occasionally detected after few months of therapy.

Limitation of our study was that we did not use any provocative testing for the assessment of the HPA axis as the study design was retrospective. However, studies have shown that serum morning cortisol levels under approximately 100 nmol/l are suggestive of adrenal insufficiency [11,22]. Our routine cutoff of 69 nmol/l by clinical laboratory was even stricter. Hypocortisolaemia at this level can represent only the tip of the iceberg of the HPA axis dysfunction. Patients that have serum morning cortisol values within the reference range in normal circumstances might still have an inadequate adrenal response to stress [12]. Therefore it is more than likely that our results underestimate the prevalence of adrenal insufficiency and that some degree of HPA axis suppression is present in more than 20% percent of paediatric IBD patients at the end of systemic glucocorticoid course. The cortisol levels, however, were measured by request of clinicians and not on all patients treated with glucocorticoids during this time period.

Potential confounding factors that could affect the serum morning cortisol measurement are the cross-reactivity of the steroid preparations with cortisol assays, timing of the blood sample, compliance to the prescribed medication and possible stress factors that could elevate the serum cortisol levels, such as infections. Most of the common glucocorticoid drugs cross-react with cortisol assays. Therefore, our patients were advised not to take their daily steroid dose on the sample day and the sample was scheduled to be withdrawn at 9:00, approximately 24 hours after the last steroid dose. At that point, no excess glucocorticoid activity (i.e. drug induced activity) from the ingested medication can be measured from the sample and this approach was also recommended in the international guidelines [17,23]. The compliance of the patients to the medication is a frequent matter of debate. Most of the paediatric IBD patients, however, seem to adhere well to the medication as demonstrated in our study on measured glucocorticoid bioactivity in serum [23]. In addition, none of the patients presented with fever and/or symptoms of infection likely to cause a stress response.

## Conclusions

In conclusion, this study shows that at least every fifth paediatric IBD patient presents with abnormal or even undetectable serum cortisol values at the end of systemic glucocorticoid treatment. Without screening, however in many patients the condition goes unnoticed. For the patients with low levels of cortisol, hydrocortisone substitution was introduced until observing cortisol values within normal range. This practice reduces the risk of serious consequences of adrenal insufficiency [14] and most likely improves the wellbeing of the patients. We recommend screening for adrenal insufficiency in children with IBD when the daily dose is low and/or the therapy is about to be discontinued to depict patients in need of hydrocortisone substitution.

## Abbreviations

ACTH: Adrenocorticotropic hormone; 5-ASA: 5-acetosalicylic acid; Aza: Azathioprine; CD: Crohn’s disease; HPA axis: Hypothalamic-pituitary-adrenal axis; IBD: Inflammatory bowel disease; IBDU: Unclassified colitis; UC: Ulcerative colitis.

## Competing interests

The authors have no financial, professional or personal conflicts of interest relevant to the manuscript.

## Authors’ contributions

MS participated in the planning of the study concept and design, analyzed the data and drafted the manuscript. KLK supervised the study, obtained funding and critically revised the manuscript. Both authors read and approved the final manuscript.

## Pre-publication history

The pre-publication history for this paper can be accessed here:

http://www.biomedcentral.com/1471-230X/14/51/prepub
